# What’s needed to sustain malaria elimination in the Region of the Americas

**DOI:** 10.2471/BLT.23.290506

**Published:** 2023-10-01

**Authors:** Maria Eugenia Grillet

**Affiliations:** a Laboratorio de Biología de Vectores y Parásitos, Instituto de Zoología y Ecología Tropical, Facultad de Ciencias, Universidad Central de Venezuela, Avenida Los Ilustres, Los Chaguaramos, Caracas 1041-A, Venezuela.

Eliminating malaria remains one of the world’s greatest public health challenges. The World Health Organization (WHO) has set the ambitious goal of reducing malaria mortality and morbidity by 90% by 2030.^1^ Across the WHO Region of the Americas, malaria was common, but it was eliminated from the United States of America, the Caribbean and most of Venezuela throughout the 20th century.^2^ The burden of disease in the Region has been decreasing, particularly from 2000 to 2015, when the incidence of cases declined by 62% and malaria-related deaths by 61%.^1^ In the last four years, WHO has certified four countries as free of malaria, and eight more are on track to eliminate the disease by 2025.^1^ However, despite progress, malaria remains endemic in 18 countries across the Region, where 139 million people are at some risk of infection^1^ and where the risk of the resurgence of the disease in the malaria-free countries is a constant threat. For example, Jamaica and Trinidad and Tobago had outbreaks of *Plasmodium vivax* and *P. falciparum* after 44 and 25 years of successful elimination, respectively^3^ – outbreaks both countries managed to control. In Venezuela, malaria due to *P. vivax* and *P. falciparum* re-emerged in the early 1980s and the trend continues, making Venezuela the country with the highest malaria morbidity and mortality in the Region.^1^^,^^4^ Furthermore, recent outbreaks of locally acquired *P. vivax* malaria in the United States demonstrate the ongoing risk of disease reintroduction in non-endemic areas.^5^

Malaria elimination involves interrupting transmission in a country until no parasites remain, while eradication is elimination on a global scale. Thus, after a country becomes malaria -free, the importation of malaria is a persistent threat. Both humans and mosquitoes from endemic regions can introduce the parasite at any time. Although malaria was eliminated in the United States in the early 1950s, the country has recorded five autochthonous outbreaks since 2000. These outbreaks were expected events since competent *Anopheles* vectors still exist, seasonal local climatic conditions are suitable for the development of *Plasmodium,* and the country receives a high number of travellers from endemic areas.^6^ Over the past decades, the reported number of imported malaria cases has steadily increased,^7^ suggesting that local malaria outbreaks will continue to pose a health threat in the United States. Malaria-free countries are advised to maintain the ability to detect and control imported malaria until eradication is achieved, either by maintaining continuous vector control measures and/or by robust and intensive surveillance to identify, treat and cure all imported infections promptly, and to avoid a resurgence of malaria transmission. Paradoxically, many successes in malaria control have historically led to a relaxation of health efforts and a setback in progress in the fight against the disease.^8^ Post-elimination control measures must continue, and local and regional funds should be allocated to this end. 

The impact of climate change on increasing the suitability of malaria transmission is documented,^9^ but little is found on how this impact might reverse elimination successes. A climate-related event could promote more frequent contact among imported *Plasmodium*, the local vectors, and persons with low immunity to malaria in countries that are no longer malaria endemic. Storms, heavy rainfall and floods will create stagnant water, increasing breeding grounds for *Anopheles* mosquitoes.^10^ Rising temperatures could expand the suitable environment for malaria transmission, further exposing populations with low immunity to imported malaria infections. In addition, an upsurge in the frequency of weather-related disasters or regional climate variability could increase the risk of malaria reintroduction in malaria-free countries. The health of the population in Central and South America is particularly affected by climate change, a vulnerability amplified by inequality, poverty, high population density, deforestation and the intense degradation of land. The Region is also an area sensitive to climate-related migrations and displacements due to droughts, tropical storms, heavy rains and floods,^9^ which could result in people with malaria moving to areas where malaria is no longer endemic. 

To successfully safeguard the gains made in the fight against malaria, the countries of the Region need resilient health systems capable of preparing for and responding effectively to the impact of increased frequency and exposure to climate-sensitive diseases such as malaria. Climate services such as climate forecasts and projections should be strengthened or incorporated and integrated into malaria surveillance to better respond to the increased risk. Political, institutional and financial barriers must be overcome to implement strong health systems able to face current and future public health challenges, and to sustain the elimination of malaria in the Americas.
